# Nutritional Strategies against Diabetic Nephropathy: Insights from Animal Studies and Human Trials

**DOI:** 10.3390/nu16121918

**Published:** 2024-06-18

**Authors:** Jiayi Zhou, Nora Franceschini, W. H. Davin Townley-Tilson, Nobuyo Maeda-Smithies

**Affiliations:** 1 Department of Nutrition, Gillings School of Global Public Health, University of North Carolina at Chapel Hill, Chapel Hill, NC 27599, USA; jz0105@email.unc.edu; 2 Department of Epidemiology, Gillings School of Global Public Health, University of North Carolina at Chapel Hill, Chapel Hill, NC 27599, USA; noraf@unc.edu; 3 Department of Pathology and Laboratory Medicine, School of Medicine, University of North Carolina at Chapel Hill, Chapel Hill, NC 27599, USA; davin_townley_tilson@med.unc.edu

**Keywords:** nutrition, diabetic nephropathy, metabolism, autophagy, antioxidant, gut microbiome

## Abstract

Diabetic nephropathy (DN), defined as continuously elevated urinary albumin and a diminished estimated glomerular filtration rate, is a serious complication of both type 1 diabetes and type 2 diabetes and is the main cause of end-stage kidney disease. Patients with end-stage renal disease require chronic kidney dialysis and/or a kidney transplantation. Research highlights the role of diet in modulating specific signaling pathways that are instrumental in the progression of DN. Nutrient-sensitive pathways, affected by nutritional compounds and dietary components, offer a novel perspective on the management of DN by influencing inflammation, oxidative stress, and nutrient metabolism. Animal models have identified signaling pathways related to glucose metabolism, inflammation responses, autophagy, and lipid metabolism, while human population studies have contributed to the clinical significance of designing medical and nutritional therapies to attenuate DN progression. Here, we will update recent progress in research into the renoprotective or therapeutic effects of nutritional compounds, and potential nutrition-modulated pathways.

## 1. Introduction

Nutrition plays a pivotal role in both the development and management of diabetic nephropathy (DN), a severe complication of diabetes characterized by progressive albuminuria, declining glomerular filtration rate, podocyte loss, and renal fibrosis [1]. Decades of studies have pointed out that nutritional components are integral to the etiology and progression of DN, primarily through the disturbance of lipid absorption and metabolism, the induction of oxidative stress secondary to hyperglycemia, and the propagation of inflammatory processes [2]. These pathophysiological mechanisms collectively contribute to renal impairment, manifesting in both structural and functional deterioration of kidneys [3]. Importantly, the gut, where fibers are fermented by microbiota to short-chain fatty acids (SCFAs), has emerged as a crucial therapeutic target. SCFAs help maintain glucose homeostasis and regulate lipid metabolism, showcasing the profound impact of diet on DN [4,5]. Additionally, dietary modifications such as reducing protein intake or increasing consumption of antioxidants through plant-based nutrients have been found to help slow the progression of kidney damage and subsequent DN [6,7,8]. Therefore, the complex interrelationships of individual nutritional elements highlight the need for a greater understanding of the molecular and signaling routes implicated in DN, with specific attention given to gut–kidney axis dynamics and diet in managing patients with DN.

## 2. Mechanistic Studies

### 2.1. Nutritional Compound: Autophagy, Nutrients Metabolism, and Oxidative Stress

#### 2.1.1. Lipid Metabolism and AMPK Signaling

**Figure 1:**
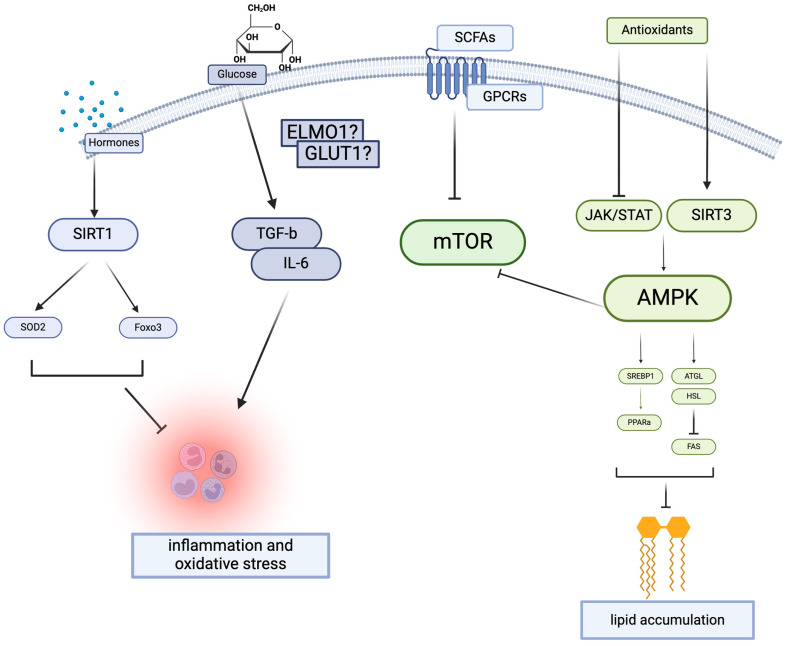
Nutrition-related signaling pathway leading to diabetic nephropathy. Created With BioRender.com.

DN is a complex disease marked by inflammation [9], oxidative stress [10], and disrupted nutrient metabolism. In the kidneys, the progression of DN is marked by lipid accumulation within podocytes and in tubular cells, leading to inflammation and functional deterioration of these cells [3,11]. The effectors of oxidative stress and metabolism are schematically described in Figure 1. Therefore, monitoring nutrition metabolism has been crucial in managing the progress of DN. For instance, insulin, which plays a crucial therapeutic role in blood glucose regulation in diabetes [12], is the central molecule of glucose metabolism and the key factor for diabetes [13]. However, numerous endogenous molecules, proteins, and hormones such as adiponectin and leptin, have been recognized for their integral roles in nutrient metabolism, their modulation of the AMP-activated protein kinase (AMPK) pathway, and their therapeutic potential in DN [14], particularly concerning lipid metabolism [15,16,17,18].

Meteorin-like (Metrnl) is a hormone secreted from muscle upon exercise and from adipose tissues upon cold exposure and it plays a role in immunoregulation and metabolism [19]. Lipogenesis gene expression was decreased in response to overexpression of Metrnl [20]. Metrnl also upregulates the Sirtuin 3 (SIRT3)–AMPK signaling axis, as Metrnl restores the SIRT3–AMPK function to protect mitochondrial dysfunction and assist in lipid clearance in renal cells [21]. Targeting AMPK activation through other proteins like junctional adhesion molecule-like (JAML) presents another promising approach. JAML, a member of the immunoglobulin family, is expressed in podocytes in the kidney [22]. Reducing JAML levels will reduce lipid levels in podocytes and contribute to DN management strategies. JAML deficiency leads to an increase in AMPK phosphorylation and greater SIRT1 expression, as both have critical roles in lipid metabolism in vivo and in vitro [22]. Furthermore, drugs that mimic hormone functions show effectiveness in modulating AMPK pathways. Liraglutide, a drug that mimics the incretin hormone and functions as a glucagon-like peptide-1 (GLP-1) receptor agonist, exhibits functions in enhancing lipid metabolism and mediating glucose balance [23]. Originally approved for weight management in patients with type 2 diabetes, liraglutide has been found to increase AMPK expression in DN-affected mice [24]. This increase in AMPK phosphorylation supports lipolysis through the activation of enzymes such as adipose triglyceride lipase (ATGL) and hormone-sensitive lipase (HSL) and decreases lipid synthesis via sterol regulatory element-binding protein 1 (SREBP-1) and fatty acid synthase (FAS) [24], resulting in a reduction in renal ectopic lipid deposition.

#### 2.1.2. Nutritional Compounds

Natural nutritional compounds also prevent DN progression by enhancing lipid metabolism [25]. Chrysin, also known as 5,7-dihydroxyflavone, is a flavonoid found in honey [26]. Chrysin aids in managing obesity and improving insulin sensitivity by altering lipid metabolism via the activation of AMPK and its downstream, such as SREBP-1 [27]. Anti-obesogenic and anti-lipogenic effects were observed when an AMPK inhibitor was administered to a rat model of DN [28]. Resveratrol, a polyphenolic compound in fruits like grapes, nuts, and berries, possesses antioxidant properties and provides multiple health benefits, including anti-inflammatory and anticancer effects [29]. Resveratrol supports kidney health by enhancing lipid metabolism through the reduction in SREBP-1, and stimulation of the AMPK/mTOR (mammalian target of rapamycin) pathway, which promotes autophagy [30]. Resveratrol is also known to activate SIRT1 in response to oxidative stress and inflammation in diabetic rats. Specifically, resveratrol-induced activation of SIRT1 activates the AMPK cascade, and leads to acetylation of the class O forkhead box (FOXO) family [31] and the production of antioxidant enzymes such as SOD2 [32]. In rats, resveratrol treatment ameliorated high-glucose-induced oxidative stress by upregulating deacetylase activity. This treatment also attenuated the high expression of acetylated-FOXO3a [31]. Additionally, SIRT1 activation by resveratrol deacetylated SOD2, an important antioxidant enzyme, further enhanced the antioxidant response in diabetic rats treated with resveratrol [32]. Sulforaphane (SFN), found in cruciferous vegetables, has also been shown to combat diabetes and inflammation [33]. SFN’s ability to activate nuclear factor erythroid 2-related factor 2 (Nrf2) and to interact with AMPK plays a role in preventing DN. Wildtype mice on a high-fat diet treated with SFN showed less renal damage than diabetic mice lacking AMPK on a similar diet, underscoring the therapeutic potential of activating Nrf2 through AMPK pathways in DN [34].

### 2.2. Gut–Kidney Axis

#### 2.2.1. Fiber Intake and Gut Microbiota

In a clinical study, an imbalance of gut microbiota has been observed in the fecal samples of patients with DN. These showed notably lower levels of butyrate and probiotics compared with diabetic patients without nephropathy [35], suggesting that disruptions in glucose homeostasis and lipid dysregulation lead to disease development [3,36].

Dietary fibers are indigestible carbohydrates which are fermented into short-chain fatty acids (SCFAs) in the presence of gut bacteria [36]. Dietary fiber composition has been known to alter the balance of the gut microbiome [35]. Diabetic mice fed fiber demonstrated a significantly reduced level of albuminuria, and renal fibrosis was correlated with an increased level of SCFAs [37]. Inulin is a type of prebiotic dietary fiber that can regulate the composition of the gut microbiome, which possibly leads to beneficial effects on the human body. A type of fiber called inulin-type fructans has been shown to help grow beneficial bacteria like *Bifidobacterium* species in the gut. These bacteria then produce SCFAs [38,39], which can help better regulate blood sugar levels and improve insulin sensitivity [37]. Sodium butyrate, another SCFA that gut microbiota produce from dietary fiber, demonstrated effects in insulin sensitivity and regulating inflammation [40,41,42], and the plasma level of sodium butyrate were positively correlated with eGFR [40,41,42]. Specifically, administration of sodium butyrate reduced the enlargement of the glomerular areas and decreased the expression of fibronectin and collagen IV in renal tissues of diabetic mice [35]. In addition, the injection also increased phosphorylation of AMPK and decreased phosphorylation of mTOR in renal tissue, suggesting enhanced energy metabolism [35]. Altering the microbiota with fiber treatment has been effective, suggesting that precision nutrition aimed at changing gut bacteria can be a promising approach to slowing the progression of DN.

Uremic retention solutes are compounds that accumulate in the blood when kidney excretory function declines. When these solutes are elevated, some can be considered as uremic toxins [43]. The gut microbiota can be a source of uremic toxins, which can contribute to the progression of renal conditions, including DN [44]. Phenyl sulfate (PS) is an untargeted gut-microbiota-derived metabolite, which is correlated with the increase in albuminuria in diabetic patients [44]. In STZ-induced diabetic mouse models, diabetic mice treated with PS showed significant change in plasma PS levels after 6 weeks, along with podocyte damage and elevation in inflammatory genes including *Tgf-β*, potentially developing renal fibrosis [44]. Multiple human-level studies have demonstrated that indoxyl sulfate levels are positively related to multiple renal biomarkers and negatively correlated to renal functions [45,46,47]. Also, the serum p-cresol value was elevated in diabetic patients, which was associated with declined renal function [48]. However, there is limited evidence from animal studies of the potential therapeutic effects of removing or limiting indoxyl sulfate or p-cresol production with the aim of ultimately restoring kidney functions [49,50].

#### 2.2.2. SCFA Cellular Receptors

On the cellular level, SCFAs’ signals through GPCR are also being studied. Hydroxycarboxylic acid receptor 2, also known as GPR109A, is a G-protein-coupled receptor involved with the signal of SCFA butyrate, and free fatty acid receptor 2 (called FFAR2 or GPR43) is another receptor that responds to acetate [51]. When treated with butyrate and acetate, respectively, knockout of GPR109A and GPR43 showed moderate improvement of albuminuria. The protective effect with loss of GPCRs was not fully demonstrated, as indicated by albuminuria [51]. The clinical approach to the use of gut microbiota is fecal microbiota transplantation, which is gaining popularity for its ability to manage lipid metabolism and, consequently, renal health in diabetes [52]. Diabetic mice that received fecal microbiota extracted from a non-diabetic group showed a decrease in levels of serum IL-6, an inflammatory biomarker. The transplanted group also showed an improvement in necrosis of tubular epithelial cells, indicating the effectiveness of the transplantation in improving renal injuries [53].

### 2.3. Vitamin D: Autophagy and Metabolism

Vitamin D plays a crucial role in improving conditions associated with DN through various mechanisms [54]. In the kidneys, vitamin D preserves podocyte health [54], reduces renin gene expression [55], and decreases markers of inflammation [56,57]. The biologically active variant of vitamin D, known as 1,25-dihydroxyvitamin D3 (calcitriol), binds to the vitamin D receptor (VDR) in podocytes [58,59]. Activation of VDR initiates a broad spectrum of biological responses, encompassing both genomic regulation, such as VDR gene-binding capacity in the nucleus [60], and non-genomic regulation of signaling cascades, such as AMPK pathways and signal transducers and activators of transcription (STAT) signaling [61]. The nutrients related pathways to the activation of autophagy and lipid metabolism are illustrated in Figure 1. Podocyte autophagy, a process for degrading damaged proteins like albumin, is a natural protective mechanism. Individuals with diabetes exhibited elevated urinary albumin and serum urea [62], as VDR overexpression can mitigate defective podocyte autophagy in DN via AMPK [63]. With vitamin D treatment, both urinary albumin and serum urea levels were diminished, and the production of inflammation-related cytokines, such as IL-6 and TNF-alpha, was downregulated [64]. Control mice displayed a significant presence of VDR in the nuclei of glomerular podocytes, while VDR levels were decreased in the DN group. Treatment with calcitriol reduced albuminuria, improved the condition of kidney tissue, enhanced the health of podocytes, and preserved autophagy function within these cells [64]. Administration of calcitriol to diabetic mice resulted in the activation of autophagy and increased VDR expression in the DN group compared with controls. Calcitriol treatment also mitigated renal failure and podocyte injury, showing a reduction in podocyte autophagy [61]. Together, these results indicate that VDR and active vitamin D3 treatment might be potential therapeutic routes for DN.

Mammalian target of rapamycin (mTOR) is a key regulator of cellular response to nutrients and growth factors [65,66]. Diabetic mice treated with vitamin D exhibited a significant decrease in mTOR expression in the kidney compared with the DN group. These results suggest that reduced mTOR activity in podocytes safeguards podocytes and proximal convolute tubule cells, then slows down DN progression using vitamin D [61], and serves as a viable therapeutic approach to avert DN. Progressive glycemic control of renal dysfunction results in the uncontrolled generation of reactive oxidative species (ROS) [2]. High glucose levels significantly increased ROS in glomerular mesangial cells, which was partially countered by vitamin D supplementation [57]. High glucose and ROS led to the activation of the janus kinase (JAK) and STAT pathway demonstrating that vitamin D can significantly reduce the activity of this pathway, as evidenced by decreased levels of phosphorylated JAK2. These results show that vitamin D suppresses the activation of the JAK/STAT cascade induced by high glucose, presenting it as a potential option for treating DN [57].

A vitamin D analog, 22-oxacalcitriol (OCT), significantly decreased mean arterial blood pressure, kidney weight index, serum creatinine, blood-urea nitrogen, and urinary albumin excretion, and improved creatinine clearance in the DN group compared with untreated DN rats [67]. OCT treatment led to improvements in renal health and glucose homeostasis, which were associated with enhanced autophagy and regulated cytokine levels [67]. High glucose levels contribute to the activation of TGF-β, which subsequently stimulates the formation of matrix proteins [53]. TGF-β levels are significantly reduced by treatment with vitamin D, and this suppressive effect of vitamin D treatment is hindered when VDR is silenced using siRNA, thereby mitigating the inhibitory mechanism of vitamin D [68]. Despite showing effectiveness in mouse models, there is a lack of evidence for AMPK signaling and vitamin D receptor activation to be effective treatments for diabetic nephropathy in humans [69]. Treatment with calcitriol led to reduction in albuminuria levels, but the results were not significant compared to untreated patient groups [69,70,71]. Multiple studies have demonstrated the deficiency of calcitriol in diabetic patients, and a lower level of vitamin D is associated with a higher risk of DN and a higher prevalence of renal impairments [72,73,74]. Although multiple randomized control trials have revealed a potential causal relationship between low vitamin D status and DN [75,76], there is still no concrete evidence indicating the effectiveness of vitamin D treatment in decreasing the DN risk and slowing DN progression [69].

Mechanistically, targeting the AMPK signaling pathway shows promise in reducing lipid deposition and enhancing metabolism, suggesting the potential for less invasive treatments of activating endogenous molecules to decrease lipid accumulation in the kidneys. The targeted interventions are systematically organized in Table 1 through pathways, models, and potential effects on renal functions. The integration of AMPK activators with established therapies like Metformin and ACE inhibitors, as well as treatments addressing inflammation and oxidative stress, is worth further investigation [77]. Exploring the microbiota’s role through fiber treatment has shown the effectiveness of precision nutrition in altering gut bacteria, with a focus on GPCR activities. More research can also focus on resolving gut-derived renal toxins as a means to maintain renal functions. Furthermore, there is potential for enhancing VDR in podocytes and leveraging vitamin D therapies to target the mTOR pathway. This indicates valuable research directions in both nutrient-based and molecular-targeted therapies for managing DN.

## 3. Human Level Trials

### 3.1. Protein Restriction on DN Progression

In clinical practice, medical nutrition therapy for patients with chronic kidney disease often includes protein restriction. The most recent 2024 Kidney Disease: Improving Global Outcomes CKD work shop (KDIGO) recommends that patients with CKD at stages 3 to 5 who are not on dialysis maintain a protein intake of 0.8 g per kilogram of body weight per day [78]. This is an increase from the previous guideline in the National Kidney Foundation’s Kidney Disease Outcomes Quality Initiative (KDOQI) which recommends 0.55 to 0.6 g per kilogram of body weight, set in 2020 [79]. The updated recommendation aligns with the general population’s recommended protein intake of 0.8 to 1.0 g per kilogram of body weight. Specifically, for patients with CKD who have diabetes, the recommendation is to maintain a 0.6–0.8 g per body weight of protein intake to stabilize nutritional status and glycemic control [80].

Animal studies have indicated that in comparison with a conventional or unrestricted protein diet, a high protein diet can result in elevated intraglomerular pressure and glomerular hyperfiltration, consequently harming the glomerular architecture [81,82]. However, recent evidence also suggests that the disadvantages of a low-protein diet, such as malnutrition, may outweigh its benefits for protecting kidney function [83], which leads to further consideration of using protein restriction in DN medical nutrition therapy. There is a scarcity of research specifically providing protein intake guidelines for patients with DN [84,85,86]. There is inconclusive evidence regarding the efficacy of a low-protein diet in decelerating the decline of glomerular filtration rate in DN patients [87]. Additionally, a study of protein-restricted diets showed there was little to no effect on urinary albumin excretion (UAE) and eGFR in patients with type 2 diabetes [88].

Altogether, protein restriction alone exhibits uncertain renoprotective effects. Many patients need more protein intake to sustain the body’s normal metabolic functions and system regulation [89]. On the other hand, intervention using flour with high-resistant starch and low protein content markedly stabilized blood glucose and lipid profiles, reduced serum uric acid and urinary beta-2 microglobulin (β2-MG) levels, and bolstered antioxidative stress defense [90]. Similarly, when comparing the effects of Mediterranean-diet protein sources like low-fat dairy, fish, poultry, soy, and legumes, against Western-diet protein sources such as red and processed meats, eggs, and high-fat dairy, the Western dietary pattern was associated with increased DN risk, and diets high in animal protein and low in fruits, vegetables, and fiber had adverse effects on kidney health [91]. Replacing red meat with chicken or plant protein was associated with reduced urinary albumin excretion, implicating the role of lipid metabolism in renal health and cholesterol management [92].

### 3.2. Antioxidants: Polyphenols and Flavonoids

Excessive oxidative stress can trigger inflammation and cytokine, chemokine, and NF-κB pathway activations, ultimately leading to kidney damage [93]. The composite dietary antioxidant index (CDAI), which measures intake of six food-sourced antioxidants—selenium, zinc, vitamins A, C, and E, and carotenoids—from 24 h dietary recalls—was used to assess the dose-responsive effects on DN [94]. Higher CDAI levels were independently associated with lower risk factors for DN. Thus, maintaining a diet rich in antioxidants, as indicated by higher CDAI levels, can decrease the incidence of kidney disease among patients with diabetes, and mortality in different stages of DN.

Dietary polyphenols have gained attention for their roles in reducing the risk of diabetes and its associated complications [95]. Grapevines and berries are rich in resveratrol, a polyphenol that has been utilized as a dietary supplement for its various health benefits, including kidney protection through multiple mechanisms [96]. First, resveratrol can interfere with the signaling pathways of ROS production and safeguard the kidneys from damage caused by oxidative stress in diabetes by enhancing the antioxidant defense system and reducing lipid peroxidation via the AMPK pathways [96]. Another significant function of resveratrol is to stimulate autophagy through the inhibition of podocyte apoptosis where autophagy activation is considered a strategic approach for DN treatment [22]. Furthermore, treating mice with a high-fat diet with resveratrol notably enhanced renal health as it significantly reduced the proliferation of kidney fibroblast cells under high glucose conditions by activating AMPK and ROS overproduction [97,98]. Flavonoids also showed effects in ameliorating kidney injury through various mechanisms such as regulation of the RAS, reduction in oxidative stress, promotion of anti-inflammatory effects, and modulation of lipid and glucose metabolism [99]. In a human trial, the higher intake of flavan-3-ols and flavones was significantly linked to a lower progression rate of DN [100].

### 3.3. Salt Intake

In the initial phases of DN, the assessment of estimated daily salt consumption is infrequently conducted in routine clinical settings. Reducing salt intake has been recognized as a significant factor in mitigating hypertension and vascular disease, and slowing kidney disease progression in diabetes [101]. Lowering salt intake can reduce blood pressure both in the short term and over a longer duration, decreasing UAE, although no significant changes were noted in eGFR or HbA1c levels [101]. UAE decreases in patients implies that meticulous management of salt intake is likely more beneficial for patients with chronic kidney disease with stages 2 and 3 of DN [102]. This suggests that while salt restriction contributes to managing DN, it is not sufficient as a standalone dietary approach; additional vascular factors must also be considered.

Treatment for high blood glucose could be performed under different salt consumption levels. For example, researchers measured foundational urinary sodium to creatinine ratios (UACRs) to understand the impact of intervention with type 2 diabetes and albuminuria [103]. Dapagliflozin, an anti-diabetic medication, was linked to a reduction in albuminuria irrespective of baseline salt consumption. As a sodium/glucose cotransporter 2 (SGLT2) inhibitor, this drug reduces glucose reabsorption in the kidneys and improves insulin sensitivity. Tamura and his colleagues illustrated that dapagliflozin’s effect on reducing eGFR from baseline was more pronounced in the high-sodium group compared with the low-sodium group, more likely related to improvement in glomerular hyperfiltration, coupled with the add-on effects of SGLT2 inhibitor. Thus, it highlights the importance of considering dietary intake when prescribing medicine such as natriuresis-related hemodynamic effects of SGLT2 inhibitors in DN management [103].

### 3.4. Omega-3 and Omega-6 Fatty Acids

Previous studies indicated the protective role of omega-3 fatty acids on DN progression, focusing on the effects of EPA and DHA on kidney function and metabolic biomarkers in diabetic patients [104]. While omega-3 supplementation was associated with reduced proteinuria and higher eGFR compared with control subjects, these differences were not statistically significant to reach a conclusion about the intake of omega-3 fatty acids and its association with the development of DN [105]. A weak correlation was observed among the ratios of eicosapentaenoic acid (EPA) to arachidonic acid (AA), the sum of EPA and docosahexaenoic acid (DHA) to AA, and the omega-6 to omega-3 ratio with UAE. In contrast, consumption of EPA and DHA has shown a reduction in UAE in type 1 diabetes, suggesting that omega-3 fatty acids might help lower UAE and aid in managing DN. Furthermore, in type 2 diabetic individuals with hypertriglyceridemia, omega-3 fatty acid supplementation was associated with decreased UAE and reduced renal function decline [105]. Additionally, linoleic acid has been suggested to prevent type 2 diabetes onset, and a lack of alpha-linolenic and linoleic acids has been linked to DN development, indicating that omega-6 fatty acids can also benefit DN management [106]. Omega-3 fatty acids, such as EPA and DHA, show promise in improving kidney health in diabetic patients, although definitive conclusions have yet to be reached. Further research at the mechanistic level is essential to clarify the underlying pathways and to develop more precise guidelines for using essential fatty acid supplements in the treatment of DN.

In summary, effective management of DN requires a multifaceted dietary approach, focusing not only on protein intake, but also on the quality and source of protein and other dietary components. Comparative overview of observational studies and clinical trials talked in this section are summarized in Table 2. Protein-restricted diets need further research to determine the most effective administration methods, tailored to individual disease progression rates. Additionally, the role of omega-3 fatty acids like EPA and DHA in improving kidney health looks promising, though more in-depth studies are needed to establish definitive guidelines and understand the underlying biological mechanisms. Alongside these factors, antioxidant intake and considerations of other dietary elements like salt and vascular factors are crucial. Overall, a comprehensive and detailed dietary strategy is necessary to slow DN progression and enhance patient outcomes.

## 4. Conclusions

The intricate link between diabetic nephropathy and dietary nutrient intake underscores a critical avenue for both intervention and management of this progressive disease. As described above, recent research has begun to unravel the complex mechanisms by which nutritional compounds and dietary patterns influence the pathogenesis and progression of DN. Notably, dietary modulation of signaling pathways such as AMPK and mTOR presents a promising strategy for mitigating DN advancement. The significant roles of specific nutrients and dietary patterns in regulating metabolic processes [21], inflammation [53], and oxidative stress [57] are pivotal factors in DN development. Diets rich in flavonoids [100], antioxidants [93], and specific fatty acids have shown beneficial effects [105], suggesting that nutritional therapy could complement existing medical treatments by targeting these underlying mechanisms. Additionally, the gut–kidney axis has emerged as a crucial player in DN pathogenesis, with imbalances in gut microbiota linked to adverse renal outcomes. Dietary interventions, including increased intake of dietary fiber and short-chain fatty acids [35], can positively impact gut health and, subsequently, renal function.

To offer a more structured approach to managing DN, we propose leveraging these data to formulate nutritional strategies rooted in the latest preclinical and clinical evidence for effective DN management. First, dietary interventions should aim to modulate signaling pathways, incorporating foods rich in flavonoids, antioxidants, and specific fatty acids that influence AMPK and mTOR pathways to slow DN progression [21]. Second, optimizing gut health by increasing intake of dietary fiber and SCFAs can improve gut microbiota balance and renal function, while further research into specific microbiota strains by targeting GPR43 and GPR109 and their metabolites and solutes in DN management continues [35]. Individualized intake and personalized medical nutrition therapy are necessary in targeting gut microbiota functions in DN progression. Third, integrating genetics and diet by examining the interplay between host genetics, dietary patterns, and gut microbiome metabolites is vital. Investigating specific genes linked to DN susceptibility, such as ELMO1 [107], APOE [108], and GLUT1 [109], in conjunction with dietary factors, can provide deeper insights [110].

Future research should focus on longitudinal studies and clinical trials to solidify our understanding of how specific dietary components affect DN development and progression. Personalized nutrition strategies including a high-fiber diet and proper protein recommendations are essential in managing the DN process and achieving better clinical results in better glycemic control and reducing albuminuria levels [111]. Translational medicine in DN could revolutionize DN management and prevention [112]. Genetic background consideration can maximize drug response, going through pathways like vitamin D receptor polymorphism and AMPK signals in glucose handling [113,114]. As the field advances, the potential for dietary interventions to complement pharmacological treatments and improve patient outcomes in DN becomes increasingly apparent, marking a hopeful path towards more effective and holistic disease management strategies.

## Figures and Tables

**Table 1:** Summary of targeted interventions and their effects on diabetic nephropathy in animal models.

Metabolic Change	Pathway/Target	Model	Effects on DN
Lipid Handling	Sirt3–AMPK (Metrnl) [21] Sirt1–AMPK-SREBP1 (JAM) [22]	STZ + HFD mice, JAML KO mice	Attenuated renal injury, altered lipid synthesis
	AMPK-SREBP1c-PPARa (chrysin) [28] AMPKa/mTOR (resveratrol) [30] AKT/glycogen-Nrf2 (SFN) [34]	Various STZ mice/rats	Improved lipid metabolism, decreased lipotoxicity
Inflammation and oxidative stress	Butyrate-GPR43 (*F. prausnitzii*) acetate/GPR43 [35] Sirt1-FOXO3a/SOD2 [32,33]	GPCR43 KO mice, DM rats	Modulated inflammation, ameliorated oxidative stress, regulated cytokine expression and renal function
Gut Microbiota	Inulin-fermented SCFAs (ITFs) [37] GPR43/109A (dietary fiber) [53]	db/db mice, Gpr KO mice	Improved renal function and glucose and lipid metabolism, reduced fibrosis
Vitamin D Signaling	VDR Signaling (calcitriol, paricalcitol) [62] JAK/STAT (vitamin D) [57] TGF-β and inflammation response [44,58]	STZ-treated rats, VDR KO mice	Ameliorated proteinuria, enhanced autophagy, reduced inflammation

Abbreviations: AKT: protein kinase B, AMPK: AMP-activated protein kinase, db/db: diabetic mice, FOXO3: forkhead box O3, *F. prausnitzii*: *Faecalibacterium prausnitzii*, GPCR: G-protein-coupled receptor, GPR109A: G-protein-coupled receptor 109A, GPR43: G-protein-coupled receptor 43, HFD: high-fat diet, ITFs: inulin-type fructans, JAK/STAT: Janus kinase/signal transducers and activators of transcription, JAM: junctional adhesion molecule, JAML: junctional adhesion molecule-like, KO: knockout, Metrnl: meteorin-like protein, mTOR: mechanistic target of rapamycin, Nrf2: nuclear factor erythroid 2-related factor 2, PPARa: peroxisome proliferator-activated receptor alpha, SCFAs: short-chain fatty acids, SFN: sulforaphane, Sirt1: sirtuin 1, Sirt3: sirtuin 3, SOD2: superoxide dismutase 2, sREBP1: sterol regulatory element-binding protein 1, STZ: streptozotocin, TGF-β: transforming growth factor beta, VDR: vitamin D receptor.

**Table 2:** Comparative overview of observational studies and clinical trials on dietary interventions and renal outcomes in diabetic nephropathy.

Observational Studies:				
Nutritional Exposures	Disease Stage	Study Design	Participants	Outcomes
Dietary antioxidant intake	DN	Meta-analysis [94]	5676	Antioxidants lower kidney disease risk and mortality
Dietary flavonoid intake	DN and non-DN	Meta-analysis [100]	1949	Fewer flavonoids associated with DN progression
Salt intake	DN	Retrospective observational [102]	269	Higher salt intake increases SBP, HbA1c, and UAE
Omega-6 fatty acid intake	Stage 2 DN	Prospective cohort [105]	123	n-6 fatty acids with greater association with UAE
Basal sodium intake and dapagliflozin treatment	Type 2 diabetes	Secondary analysis of cohort [103]	86	Dapagliflozin decreases BP and eGFR in high-salt-intake group
Protein intake	Diabetes with diminished renal function	Retrospective cohort [88]	144	No change in UAE and eGFR with restricted protein diet
**Clinical Trials:**				
**Nutritional Intervention**	**Disease Stage**	**Study Design**	**Participants**	**Outcomes**
Altered salt intake	Type 1 or 2 diabetes	Meta-analysis of RCT [101]	313	No significant change in eGFR, reduced body weight, BP
Omega-3 fatty acids	Type 1 or 2 diabetes	Meta-analysis of RCTs [104]	344	No significant changes in BP, lower proteinuria
Low-protein diet	DN	Meta-analysis of RCTs [87]	486	No significant effect of protein restriction on DN
High-resistant starch and low-protein diet	Early DN	RCT [90]	75	Improved renal panel, blood glucose level
Mediterranean vs. Western diet	DN	Case-control study [92]	105	Improved in renal panel with Mediterranean diet

Abbreviations: BP: blood pressure, DN: diabetic nephropathy, eGFR: estimated glomerular filtration rate, HbA1c: hemoglobin A1c, RCT: randomized controlled trial, SBP: systolic blood pressure, UAE: urinary albumin excretion.
